# Efficacy evaluation of personalized coaptation in neurotization for motor deficit after peripheral nerve injury: A systematic review and meta‐analysis

**DOI:** 10.1002/brb3.1582

**Published:** 2020-03-03

**Authors:** TengDa Qian, Kai Qian, TuoYe Xu, Jing Shi, Tao Ma, ZeWu Song, ChengMing Xu, LiXin Li

**Affiliations:** ^1^ Department of Neurosurgery First Affiliated Hospital of Nanjing Medical University Nanjing China; ^2^ Department of Neurosurgery Jintan Hospital affiliated to Jiangsu University Jintan China; ^3^ Department of Neurosurgery Changzhou first people's Hospital Suzhou University Changzhou China

**Keywords:** functional recovery, neurotization, neurotube, peripheral nerve injury, personalized coaptation

## Abstract

**Introduction:**

Peripheral neurotization, recently as a promising approach, has taken effect in recovering motor function after damage to a peripheral nerve root. Neural anastomosis comprised of nerve conduit and neurorrhaphy participates in the nerve reconstruction. Current literature lacks evidence supporting an individualized coaptation for rescue of locomotor loss in rat subjects with paraplegia secondary to peripheral nerve injury (PNI).

**Methods:**

This meta‐analysis intends to qualify the specificity of gap‐specific coaptation in treating a paralyzed limb following PNI. We used a highly sensitive search strategy to identify all published studies in multiple databases up to 1 May 2019. All identified trials were systematically evaluated using specific inclusion and exclusion criteria. Cochrane methodology was also applied to the results of this study.

**Results:**

Twelve studies, including 349 rat subjects, met eligibility criteria. For a medium nerve defect (0.5–3.0 cm), nerve conduit was more likely than neurorrhaphy to precipitate axon regeneration and improve motor outcome of the hemiplegic limb (OR = 3.61, 95% CI = 1.80, 7.26, *p* < .0003) at 3‐month follow‐up, whereas neurorrhaphy might take its place in promoting limb motor function in a small nerve gap (<0.5 cm) (OR = 0.48, 95% CI = 0.22, 1.07, *p* < .007). For a small nerve defect, nerve conduit still demonstrated visible effectiveness in recovery of limb motion albeit poorer than neurorrhaphy (OR = 1.50, 95% CI = 0.92, 2.47, *p* < .05).

**Conclusion:**

Selective neurotization facilitates motor regeneration after nerve transection, and advisable choice of neural coaptation can maximize functional outcome on an individual basis.

## Graphical abstract

After peripheral nerve injury, motor loss can be rescued maximally by gap‐oriented anastomosis. It offers a valuable addition to literature and helps surgeons make an advisable option to specific nerve defect intraoperationally.

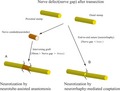

## 1 INTRODUCTION

Peripheral nerve injury (PNI), common in daily life, usually results in pronounced loss of limb movement function and places a heavy burden on healthcare system worldwide (Bao et al., 2018; Kamei et al., 2018). Historically, neurotization, a surgical approach to transfer of one nerve to another nerve, was adopted to restore a paralyzed limb after PNI, whereas discrepancy of functional outcome often resulted from an unreasonable bridge between nerve stumps (i.e., neural anastomosis or coaptaion). Recently, various biodegradable scaffolds are fabricated depended on size of the targeted nerve root, namely nerve conduit or neurotube, and applied as an intervening graft to connect a proximal stump with a distal one. Generally, after nerve transection, either end‐to‐end suture (neurorrhaphy) for a large nerve defect (nerve gap) or nerve conduit for a small one is more likely to contribute to mismatch or misdirection between regrowing axons, which thus yields limited motor outcome (Herweh et al., 2017; Texakalidis, Tora, Lamanna, Wetzel, & Boulis, 2019). Consequently, efficacious maneuvers for optimal motor regeneration are still desirable.

Of note, success for nerve reconstruction is built on the sufficient understanding of peripheral nerve regeneration pathophysiologically (Kolcun, Burks, & Wang, 2018; Yavari, Mahmoudvand, Nadri, & Rouientan, 2018). After a nerve root is severed, the proximal stump is predisposed to axonal disintergration and apoptosis while the distal stump is inclined to form disorganized mass known as neuromas. Earlier reinnervation of a denervated limb results in better motor recovery, nevertheless, nerve regeneration is known to occur at only approximately 1 mm per day (Ochiai, Matsumoto, Hara, Nishiura, & Murai, 2019; Song et al., 2019). Less time to reinnervation, distance required for regenerating fibers to arrive at end organ, and higher proportion of aligned axons between stumps are three determinants in the improvement of motor outcomes (Simic et al., 2018; Ye, Shen, Feng, & Xu, 2018). Explorations on how to elevate percentage of axonal alignment and accelerate reinnervation of the target organ are still under way.

Tension‐free neural anastomosis between stumps aids regrowth of an axotomized nerve and subserves good outcome during neurotization, whereas an overlarge gap in the damaged nerve inevitably brings a barrier to unstrained suture of nerve (Hagemann, Stucker, Breyer, & Kunkel, 2019). As a result, an intervening graft (nerve conduit) made of chitosan, less deleterious to ambient tissues, is exploited to act as a bridge between nerve stumps. Nerve conduit, physically and functionally supporting stump nerves well, plays a positive role if the nerve gap is too large to connect through direct suture in the context of an unstrained status (Frank et al., 2018). Unlike end‐to‐ end neurorrhaphy, nerve conduit also creates a microenvironment in favor of growth of regenerating axons. However, due to the reduced distance between stump nerves at the site of anastomosis, neurorrhaphy may shorten the time course of a nerve regeneration to achieve favorable outcome (Emamhadi & Andalib, 2017; Karamanos, Rakitin, Dream, & Siddiqui, 2018; Korus, Ross, Doherty, & Miller, 2016), whereas some studies indicated that misdirection and disorganizaion of axons were caused by neurorrhaphy owing to limited match between regrowing fibers (Li, Yin, Yan, Wang, & Li, 2016; Mayer, Hruby, Salminger, Bodner, & Aszmann, 2019; Socolovsky et al., 2018). Hence, whether a unique nerve defect has its own anastomosis. A brewing controversy has developed over which of this two neural coaptations is the optimal option, and no definitive guidelines exist regarding the best strategy for the treatment of motor deficit. As human applications are still in very early stage, animal models have a positive impact on the development of future conduit‐assisted neurotization. Any reliable neural coaptation will require efficacy assessment in an animal model before it can be tested in paraplegic patients. The purpose of the present study was to determine effectiveness of individualized coaptation in recovering PNI in a common laboratory rat strain, thus extrapolate the personalized anastomosis to clinic practice.

However, prospective, randomized controlled trials are needed to compare the two strategies. The studies, fabricating various nerve defects in peripheral nerve roots in rats, were reserved for illumination of the defect‐specific anastomosis. Thus, we reviewed the literature and presented a meta‐analysis of all available studies to evaluate the efficacy of personalized coaptation in the recovery of motor function in rats with PNI and clinically identify a sensible option of neural coaptation.

## 2 MATERIALS AND METHODS

### 2.1 Literature search strategy and data sources

Using PubMed, Embase, MEDLINE, cochrane library, and Web of Science database, we systematically searched the literature published up to 1 May 2019 following the PRISMA guidelines. Our search aimed to identify all original articles related to various coaptation employed in neurotization for PNI. Abstract and title search terms included were exploited for the searches. PubMed used a single term, “nerve conduit,” but Embase, and others adopted the terms “neurorrhaphy” or “direct suture” and included more specific terms for anastomosis. To be as inclusive as possible, the search also involved “anastomosis” or “anastomosis*.” The identical tactics were also used for neurotization:“neurotization” is applied in PubMed, but Embase and others use “intervening graft,” with more specific terms such as “nerve stumps’ connection” and “peripheral nerve stumps' connection.” Duplicate articles were removed, and 2 veteran reviewers screened the titles and abstracts using predetermined inclusion and exclusion criteria. If study content was unclear after reviewing the abstract, the full text was reviewed. Our search may have eliminated other studies that are not included in these databases and may be subject to publication bias. The studies, convincingly fabricating varying nerve defects on peripheral nerve roots in rats, were retained. In addition, the references of all retrieved articles were checked for additional potential studies.

### 2.2 Inclusion and exclusion criteria

The inclusion criteria were the following: (a) studies comparing effectiveness of neurotube and direct suture alone as control in rat subjects with PNI; (b) assessing functional outcome as defined by Basso, Beattie, and Bresnahan (BBB) or sciatic functional index (SFI) score (if BBB score was unavailable) at 1‐month, 2‐month, and 3‐month follow‐ups; and (c) neurotization for a peripheral nerve root injury. Exclusion criteria for our primary analysis were as follows: (a) unavailability of a neurorrhaphy comparison group; (b) unavailability of the number of subjects with functional outcome at 1‐month, 2‐month, and 3‐month follow‐ups; (c) review articles, meta‐analysis, and guidelines; (d) outcomes for allograft; and (e) nerve reconstitution for spine cord injury.

### 2.3 Data extraction and analysis

Descriptive statistics and demographic data were extracted for subjects in studies reserved. Collected data included year and country of publication, number of subjects, gender, weight, scale score of BBB and SFI, and follow‐up period. Pooled estimates of individual subject information were reported for statistical background in rats. The reported overall mean value was used in cases in which individual subject information was not available. We also collected objective outcomes of neurotization, including anastomosis in the form of nerve conduit and neurorrhaphy, with reported measurements at the final follow‐up. Data were abstracted from the eligible papers by the same 2 professional reviewers.

In some studies, allograft was also used to treat PNI other than the autograft. However, results and outcome measurements were variable. Therefore, we only studied outcomes measures in autograft. We found that both the Basso, Beattie, and Bresnahan (BBB) and sciatic functional index (SFI) scale were reported most frequently. Thus, we applied the BBB and SFI scale as outcome measures for all extracted outcomes for limb motor function. Subject outcomes were collected for neurotization involving nerve conduit and/or neurorrhaphy; outcomes for allograft were excluded in this study.

Functional outcomes in a paralyzed limb were reported for coaptation. For studies that described motor outcome as a score of SFI, we included these results as limb flexion function. The limb flexion status was also used to categorize results from 3 studies that reported motor outcome as a score of SFI. Similarly, for studies that reported motor outcome as a score of BBB, we assigned these results as extension and flexion of a paralyzed limb. Sensory recovery was not evaluated in this paper in consideration of limitation of sensory function measure. We defined motor functional recovery as reaching a minimum score of 9 in BBB or 40 in SFI for good motor outcome, with BBB score of 21 and SFI score of 100 considered as native status.

### 2.4 Quality assessment

Quality assessment for included studies was assessed by two independent reviewers. Briefly, Cochrane collaboration's tools were used for assessing quality according to the following domains: selection bias (random sequence generation and allocation concealment), attrition bias (incomplete outcome data), performance and detection bias (blinding of participants, personnel and outcome assessment), reporting bias (selective reporting), and other bias (other sources of bias). In addition, we used Newcastle–Ottawa scale (NOS) to assess the quality in the nonrandomized cohort studies.

### 2.5 Ethical approval

This research did not implicate human participants or animals.

### 2.6 Statistical analysis

The neural anastomosis was used to categorize the data: nerve conduit and neurorrhaphy. Descriptive statistics and demographic information for study participants were summarized separately. We analyzed interval data (percentage of male rats, scale score of BBB and SFI, and follow‐up period) using the Student *t* test. Rates of different approaches were compared using one‐way ANOVA, with Bonferroni correction for ad hoc comparisons. Absolute risk reductions (ARRs), odds ratios (ORs), and 95% confidence intervals (CIs) were calculated for the specified outcome. The significance of the pooled OR and ARR was determined by the *Z* test, and a *p*‐value <.05 was considered significant. The heterogeneity between studies was assessed by chi‐square‐based *Q* test and *I* ^2^ test. Heterogeneity was considered significant when *p* < .10, and pooled estimates were calculated using the random‐effects (DerSimonian–Laird) model, otherwise, a fixed‐effects (Mantel–Haenszel) model was used. Publication bias was investigated using visual evaluation of funnel plots and Egger regression asymmetry test. All statistical analyses were performed using SAS statistical software (version 9.2), Review Manager (RevMan5.3; The Cochrane collaboration) or STATA software (Peking university, Beijing, Haidian district), and probability values <.05 were considered statistically significant. The percentage of functional recovery (BBB ≥ 9 or SFI ≥ 40) for nerve conduit and neurorrhaphy was also reported.

## 3 RESULTS

### 3.1 Study and subject characteristics

**Figure 1:**
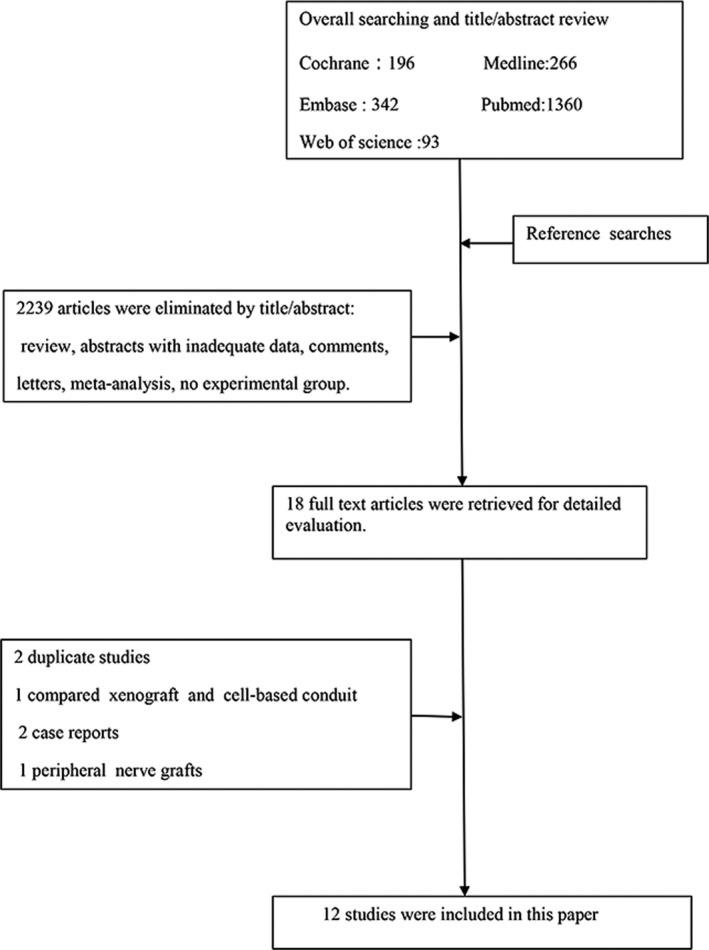
Flowchart of the literature search

The systematic methods were used to search the related databases, including the number of studies collected and excluded, and are shown in Figure 1. Overall, 12 studies met the inclusion and exclusion criteria, of which 9 were prospective studies. Studies were assigned two groups: the nerve conduit group (*n* = 175) and the neurorrhaphy group (*n* = 174). All studies assigned the scale score of BBB and SFI as functional outcomes postprocedure. The data for gaps faked individually were reserved.

**Figure 2:**
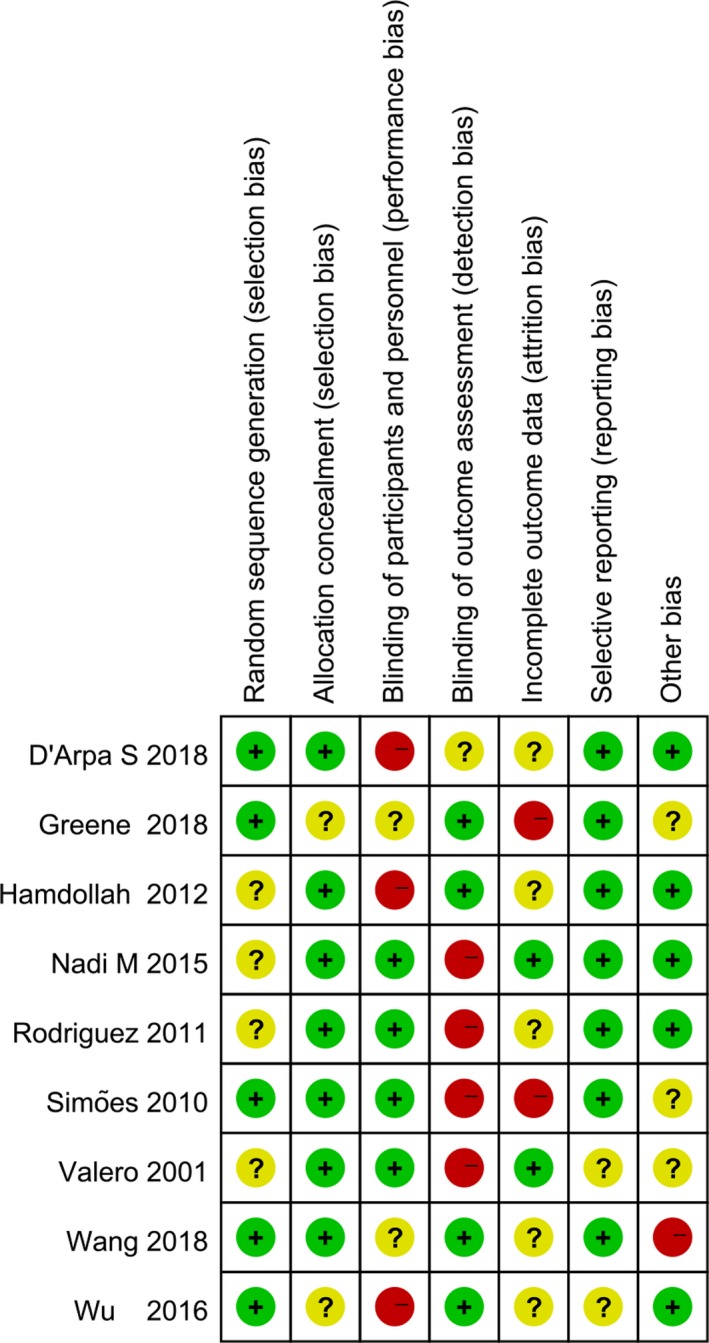
Risk of bias assessment for randomized controlled trials. “+”, low risk of bias; “−”, high risk of bias; and “?”, indicates unclear risk of bias

In this analysis, 95% of subjects were male rats, the average weight was 300 g, the mean preoperative period (interval between injury and surgery) was 2 weeks, the mean follow‐up period was 2 months (Table 1). There were no significant differences in weight, sex, or follow‐up period among rats. The key characteristics of the studies included are summarized in Table 2. Quality assessments for studies were summarized in Tables 3 and 4 and Figure 2. Briefly, for RCTs, randomization methods were described in 2 studies (Greene et al., 2018; Rodriguez et al., 2011) and allocation concealments were adequate in 1 studies. For blindness, 4 studies utilized blind observers to assess outcome, though blinded for carers were unlikely in all studies (Hundepool et al., 2018; Simoes et al., 2010; Valero‐Cabre et al., 2001; Wu et al., 2016).

**Table 1:** Summary of results included in the meta‐analysis

Study, publication year	Groups	Gap size (cm)	Male rats (%)	BBB scores	SFI scores	Mean follow‐up (months)
Rodriguez, 2011	Nerve conduit (20)	0.5–3.0 (16.80%)	19 (95%)	14 (9–15)	27 (22–34)	2
	Neurorrhaphy (20)	–0.5 (17.85%)	20 (100%)	10 (7–13)	33 (17–56)	2
Greene, 2018	Nerve conduit (17)	0.5–3.0 (15.88%)	16 (95.4%)	13 (9–16)	40 (22–55)	3
	Neurorrhaphy (17)	～0.5 (13.81%)	15 (89%)	9 (7–14)	19 (17–20)	3
Valero, 2001	Nerve conduit (6)	0.5–3.0 (6.100%)	5 (86%)	8 (6–12)	28 (22–39)	3
	Neurorrhaphy (6)	～0.5 (6.100%)	6 (100%)	6 (2–9)	25 (17–40)	3
Wu, 2016	Nerve conduit (12)	0.5–3.0 (7.58%)	11 (92%)	12 (9–17)	32 (32–50)	3
	Neurorrhaphy (12)	～0.5 (10.83%)	11 (92%)	9 (8–11)	35 (19–46)	3
Simões, 2010	Nerve conduit (14)	0.5–3.0 (12.86%)	12 (86%)	11 (9–15)	37 (28–44)	3
	Neurorrhaphy (14)	～0.5 (9.64%)	12 (86%)	10 (7–12)	41 (23–60)	3
Hamdollah, 2012	Nerve conduit (30)	0.5–3.0 (25.83%)	28 (93%)	11 (9–12)	26 (22–38)	1
	Neurorrhaphy (30)	～0.5 (24.80%)	28 (93%)	9 (8–10)	33 (20–53)	1
Wang, 2018	Nerve conduit (15)	0.5–3.0 (11.73%)	15 (100%)	13 (8–15)	18 (10–34)	1
	Neurorrhaphy (15)	～0.5 (12.80%)	15 (100%)	14 (9–17)	26 (17–34)	1
D'Arpa S., 2018	Nerve conduit (20)	0.5–3.0 (17.85%)	18 (90%)	10 (9–13)	22 (15–29)	1
	Neurorrhaphy (19)	～0.5 (14.74%)	16 (84%)	12 (8–17)	30 (14–38)	1
Cemil B., 2009	Nerve conduit (10)	0.5–3.0 (10.100%)	10 (100%)	7 (3–15)	n.r.	2
	Neurorrhaphy (10)	～0.5 (10.100%)	9 (90%)	10 (5–16)	n.r.	2
Youlai, 2015	Nerve conduit (16)	0.5–3.0 (15.95%)	13 (87%)	11 (8–15)	52 (46–63)	3
	Neurorrhaphy (16)	～0.5 (16.100%)	15 (95%)	10 (9–16)	33 (22–47)	3
García, 2014	Nerve conduit (6)	0.5–3.0 (6.100%)	5 (83%)	8 (3–10)	n.r.	3
	Neurorrhaphy (6)	～0.5 (6.100%)	4 (67%)	10 (8–13)	n.r.	3
Nadi M., 2015	Nerve conduit (9)	0.5–3.0 (6.67%)	8 (89%)	11 (8–14)	27 (22–34)	2
	Neurorrhaphy (9)	～0.5 (8.89%)	9 (100%)	13 (9–16)	33 (19–41)	2

**Table 2:** ARR and OR calculating with the corresponding 95% CI for good functional outcome following neurotization

Variables	Follow‐up	Functional outcome	Number of subjects	ARR (95%)	*p*	OR (95%)	*p*	*p*_h_
Nerve conduit Subjects in all types of defect	1‐month follow‐up	BBB > 9	79	15% (12%, 20%)	.0006	0.10 (0.05,0.40)	.003	.62
		SFI = 0	98	23% (19%, 28%)	<.001	0.14 (0.04, 0.47)	<.0001	.48
	2‐month follow‐up	BBB > 9	115	7% (−2%, 11%)	.001	0.17 (0.08, 0.30)	.004	.81
		SFI = 0	52	18% (13%, 21%)	<.002	0.21 (0.10, 0.31)	.0001	.72
	3‐month follow‐up	BBB > 9	126	3% (−5%, 6%)	.027	1.67 (1.34, 2.30)	<.0001	.51
		SFI = 0	34	12% (9%, 17%)	<.001	0.38 (0.20, 0.73)	.17	.64
Small nerve defect (<0.5 cm)	2‐month follow‐up	BBB > 9	47	16% (11%, 22%)	<.001	0.09 (0.03, 0.38)	.003	.27
		SFI = 0	38	19% (15%, 23%)	.0015	1.91 (1.20, 3.87)	<.0001	.76
Medium nerve defect (0.5–3.0 cm)	2‐month follow‐up	BBB > 9	56	18% (13%, 27%)	.057	0.68 (0.21, 2.27)	.002	.34
		SFI = 0	31	10% (9%, 16%)	<.001	0.14 (0.06, 0.35)	.0034	.36
Neurorrhaphy								
Small nerve defect (<0.5 cm)	2‐month follow‐up	BBB > 9	50	17% (14%, 19%)	.0048	1.28 (0.91, 1.73)	<.0001	.2
		SFI = 0	28	11% (7%, 18%)	<.001	0.20 (0.12, 0.35)	.27	.08
Medium nerve defect (0.5–3.0 cm)	2‐month follow‐up	BBB > 9	63	35% (30%, 43%)	.00012	0.16 (0.02, 1.10)	.06	.07
		SFI = 0	46	24% (21%, 38%)	<.001	0.33 (0.21, 0.47)	.64	.21

**Table 3:** Modified Newcastle–Ottawa Quality Assessment Scale (cohort studies)

Assessment of quality of a cohort study—Newcastle–Ottawa scale	
Selection (tick one box in each section)	
1. Representativeness of the intervention cohort	
(a) Truly representative of the HCH‐caused hemiplegia population	＊
(b) Somewhat representative of the HCH‐caused hemiplegia population	＊
(c) Selected group of participants	
(d) No description of the derivation of the cohort	
2. Selection of the nonintervention cohort	
(a) Drawn from the same community as the intervention cohort	＊
(b) Drawn from a different source	
(c) No description of the derivation of the nonintervention cohort	
3. Ascertainment of intervention	
(a) Secure record (e.g., healthcare record)	＊
(b) Structured interview	＊
(c) Written self report	
(d) Other/no description	
4. Demonstration that outcome of interest was not present at start of study	
(a) Yes	＊
(b) No	
Comparability (tick one or all boxes, as appropriate)	
1. Comparability of cohorts on the basis of the design or analysis	
(a) Study controls for nerve defects	＊
(b) Study controls for BBB: Basso, Beattie, and Bresnahan for locomotor functional recovery	＊
Outcome (tick one box in each section)	
1. Assessment of outcome	
(a) Independent blind assessment	＊
(b) Record linkage	＊
(c) Self report	
(d) Other/no description	
2. Was follow‐up long enough for outcomes to occur	
(a) Yes, if median duration of follow‐up ≥2 months	＊
(b) No, if median duration of follow‐up <2 months	
3. Adequacy of follow‐up of cohorts	
(a) Complete follow‐up: all subjects accounted for	＊
(b) Subjects lost to follow‐up unlikely to introduce bias: number lost <= 20%, or description of those lost suggesting no different from those followed	＊
(c) Follow‐up rate <80% (select an adequate %) and no description of those lost	
(d) No statement	

Note

A study can be awarded a maximum of one star for each numbered item within the selection and outcome categories. A maximum of two stars can be given for comparability.

**Table 4:** Risk of bias assessment for nonrandomized cohort studies (modified Newcastle–Ottawa scale)

First author, year	Selection	Comparability	Outcome	Total score
Rodriguez, 2011	**	***	**	7/9
Greene, 2018	**	**	**	6/9
Valero, 2001	**	**	***	7/9
Wu, 2016	**	***	**	7/9
Simões, 2010	***	*	**	6/9
Hamdollah, 2012	**	**	***	7/9
Wang, 2018	***	**	**	7/9
Nadi M., 2015	*	***	***	7/9
D'Arpa S., 2018	**	***	**	7/9

In addition, 2 studies reported rates of the follow‐up as 100% (D'Arpa et al., 2018; Wang et al., 2018), which were not mentioned in other studies. Terminally, none of studies had selective outcome reporting. For cohort studies, the results for quality assessment showed that all four studies had a moderate risk of bias (Figure 2) and reached 6–7 out of 9 points (Tables 3 and 4).

### 3.2 Neurotube‐assisted anastomosis

**Figure 3:**
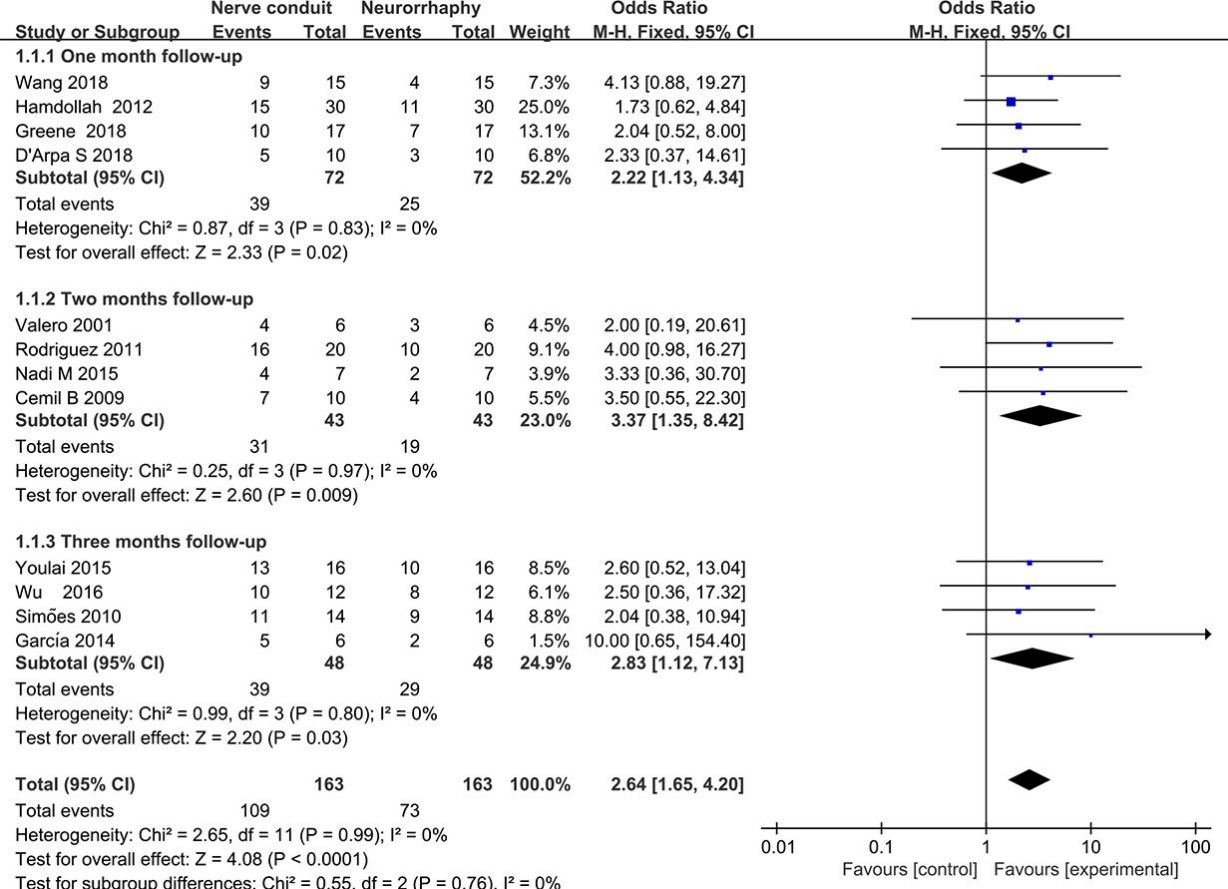
Forest plot with OR estimating with the corresponding 95% CI for favorable outcome (defined as BBB ≥ 9) associated with nerve conduit versus neurorrhaphy for individual trials and the pooled population at 1‐month, 2‐month, and 3‐month follow‐ups (subjects in all gaps). CI, confidence interval; BBB, Basso, Beattie, and Bresnahan for locomotor functional recovery; OR, odds ratio

**Figure 4:**
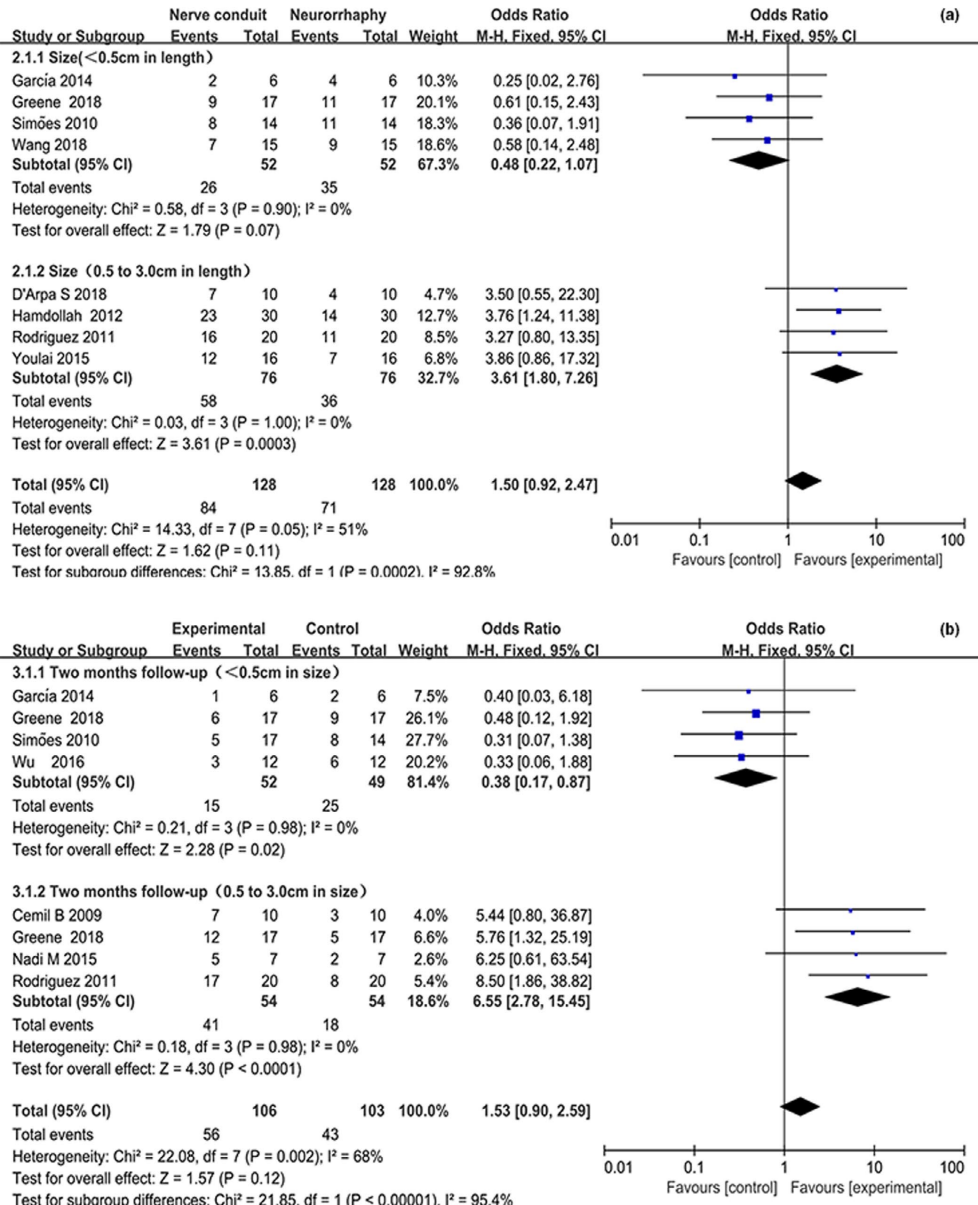
Forest plot with OR estimating with the corresponding 95% CI for (a) favorable outcome (defined as BBB ≥ 9 and SFI ≥ 40) associated with nerve conduit versus neurorrhaphy individual trials and the subgroup population stratified by size of defect for 2‐month follow‐up (b). CI, confidence interval; BBB, Basso, Beattie, and Bresnahan for locomotor functional recovery; OR, odds ratio

To recover functions of the paralyzed limbs, 175 subjects underwent neurotube‐dependent anastomosis during nerve reconstitution. Scale score of BBB or SFI was categorized as limb motor outcomes. The proportion of subjects with favorable motor outcome (recognized as BBB ≥ 9 or SFI ≥ 40) was reported for 1‐month follow‐up in 4 studies, 2‐month follow‐up in 4 studies, and 3‐month follow‐up in 4 studies. The acquired results suggested that neurotube‐assisted nerve significantly escalated number of subjects with a good outcome for 1‐month follow‐up (OR = 3.11, 95% CI = 1.72, 5.62, *p* = .002), 2‐month follow‐up (OR = 3.80, 95% CI = 2.11, 6.85, *p* < .0001), and 3‐month follow‐up (OR = 7.17, 95% CI = 3.44, 14.96, *p* < .001) (Figure 3 and Table 2). In the subgroup analysis stratified by size of a nerve defect, the number of subjects with poor functional outcome was significantly reduced in conduit‐assisted nerve transfer for 3‐month follow‐up (OR = 0.20, 95%CI = 0.08, 0.51, *p* = .0008 in the small defects and OR = 5.84, 95% CI = 2.37, 14.42, *p* = .0001 in the medium defect) (Figure 4a and Table 2). Moreover, no significant between‐study heterogeneity was detected in either subgroup or overall analysis (*p*_h_ = 0.42 for all comparisons).

### 3.3 End‐to‐end neurorrhaphy

**Figure 5:**
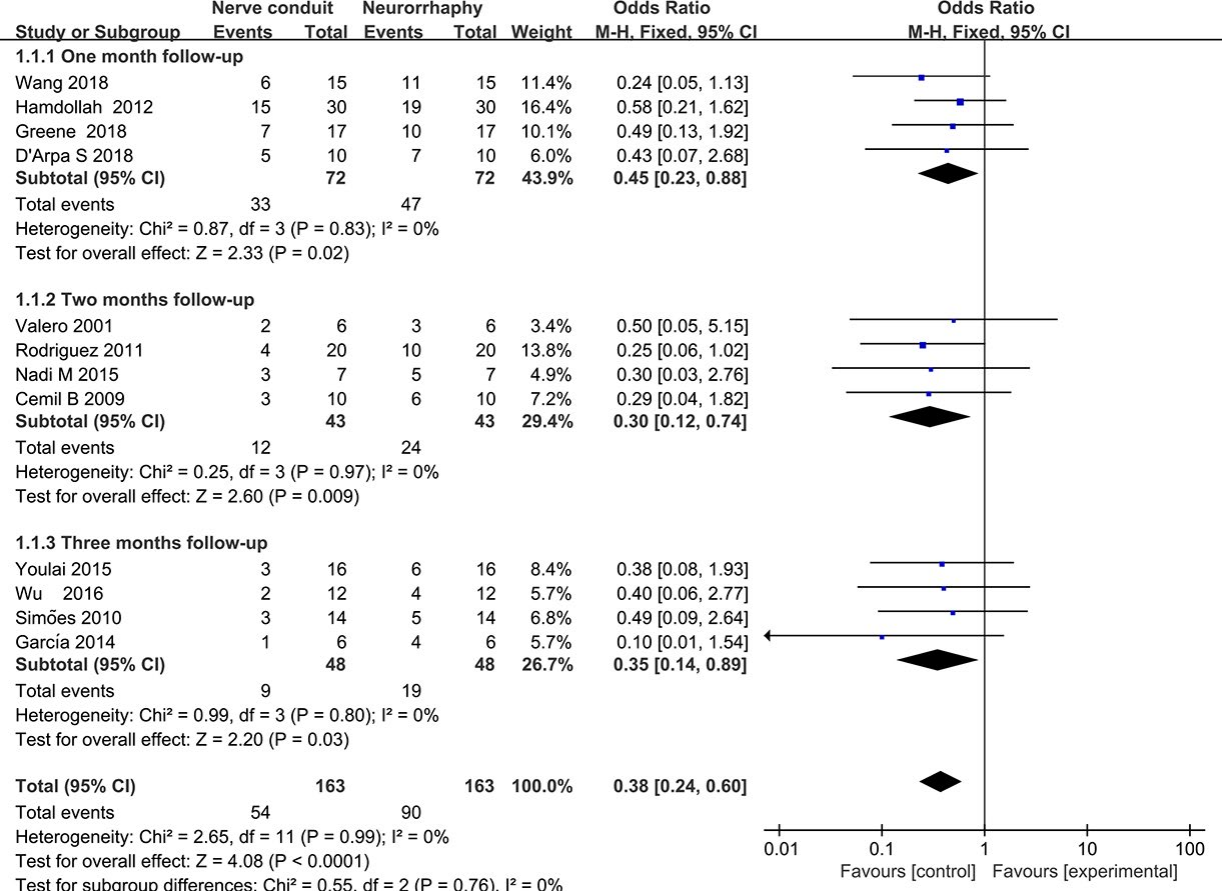
Forest plot with OR estimating with the corresponding 95% CI for the proportion of rats with poor outcome in motor function (defined as BBB < 9 or SFI < 40) associated with nerve conduit versus neurorrhaphy for individual trials and the pooled population at 1‐month, 2‐month, and 3‐month follow‐ups (rats in all defects). CI, confidence interval; BBB, Basso, Beattie, and Bresnahan for locomotor functional recovery; OR, odds ratio; SFI, sciatic functional index

A total of 6 studies involving 87 subjects were eligible for calculation of the efficacy of direct suture in activities of paralyzed limb in subjects with small nerve gap, while analogous analysis in 87 subjects with medium nerve gap was carried out. We defined scale score of BBB or SFI as a motor regeneration for hemiplegic limb. Similar results were observed in nerve gap <5 mm (OR = 0.44, 95% CI = 0.19, 1.03, *p* = .06) and in nerve gap between 5 and 30 mm (OR = 4.96, 95% CI = 2.01, 12.19, *p* = .0005; Figure 4b). The results from neurorrhaphy group were suggestive that dexterous limb motion was early regained due to an aggressive axon arrangement. (OR = 0.20, 95% CI = 0.08, 0.58, *p* = .0008). Heterogeneity was not observed in all comparisons except for in one comparison (*p*_h_ = 1.15), in which a random‐effects model was used (Figure 4). The results of pooled analysis demonstrated that conduit‐dependent nerve transfer strongly promoted reinnervation of denervated limb and sharply elevated activities of hemiplegic limbs (Figure 5; OR = 3.35, 95% CI = 1.99, 6.63, *p* < .00001).

### 3.4 Functional outcomes summary

The outcome summary for the different anastomosis strategies as categorized by BBB and SFI (defined as a flexion or extension of limb) is shown in Table 4 (Figure S1). All results were used for comparison. Two joint movement in the limb was normalized to a BBB scale score of 9 or SFI of 40 as a good outcome, full power score to a score of 21 in BBB or 100 in SFI, and no movement to a score of 0.

### 3.5 Assessment of publication bias

**Figure 6:**
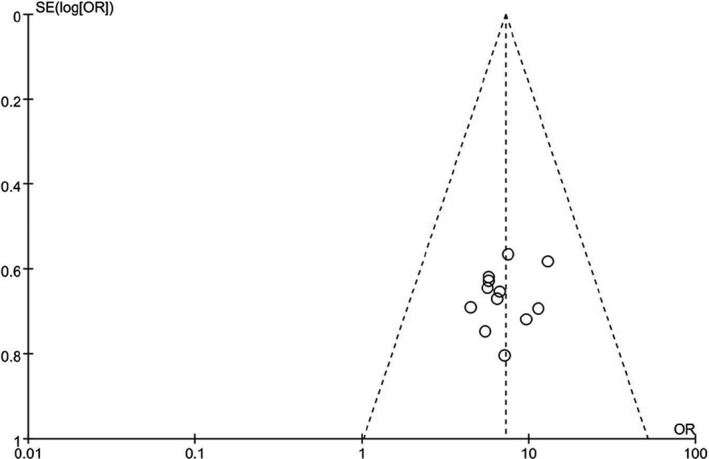
Funnel plot to detect publication bias. No significant funnel asymmetry was observed which could indicate publication bias (*p*‐value for Egger test was .27). logOR, Natural logarithm of the OR; *SE* of logOR, standard error of the logOR

Publication bias was estimated by funnel plots and Egger's test (Cemil, Ture, Cevirgen, Kaymaz, & Kaymaz, 2009). Morphology of funnel plot did not reflect obvious asymmetry (Figure 6). Then, Egger's test was used to provide statistical evidence of funnel plot symmetry, which did not show any evidence of publication bias (*p* > .18 for all comparisons), indicating that our results are statistically robust.

## 4 DISCUSSION

Peripheral nerve injury (PNI) usually occurs in 13–20 of every 100,000 persons, often alongside other damage (Garcia‐Medrano et al., 2017; Yu et al., 2015). It also frequently concerns young active persons, for whom even a partial loss of nerve function can entail serious social and economic consequences. Neurotization is considered to be one of the primary treatment options to restore limb function in patients with PNI, with active pathway reconstructed by bridging a distal stump nerve. However, owing to an oversize nerve defect, poor functional outcome frequently occurs postoperatively. As is well known, tension‐free coaptation can offer an opportunity for autonomous axonal arrangement and lead to maximal alignment of regenerating axons (Nadi et al., 2018).

Furthermore, more attention to flaccid neural coaptation has been paid by surgeons. It is reported that since an accurate axonal arrangement comes true under no strained status, a variety of nerve conduits made from advanced biodegradable materials, including laminin and fibronectin (well‐known as extracellular matrix, ECM), are applied to neural connectivity when the nerve gap is too large to carry out nerve reconstitution. On the whole, rapid regrowth and exact arrangement among axons are efficiently precipitated by ECM‐containing conduit characterized by porosity in lumen. Direct suture may be restricted to small nerve gaps in PNI and is liable to mislead axons regeneration. Consequently, neurotube‐assisted neurotization offers a vital source to recover the paralyzed limb in comparison with neurorrhaphy.

In this review, disparate nerve gaps mimicked individually in rats were used to compare the effect between neurotube and direct suture on repair by tubulization or neurorrhaphy of the peripheral nerve injury and were categorized as small nerve defect and medium nerve defect. Individualized neural anastomosis was conducted to achieve a maximal motor regeneration for the paralyzed limb. Nerve conduit‐dependent neurotization significantly increased both flexion and extension of the paralyzed limbs in subjects with medium nerve defect, compared with neurorrhaphy (*p* < .05). Instead, improvement in limb activities in subjects with small nerve defect was directly obtained from neurorrhaphy. The results were suggestive that conduit‐dependent reconstruction was likely superior to end‐to‐end suture in recovering flexion and extension for the paralyzed limbs. In our study, we assigned good outcome as BBB ≥ 9 or SFI ≥ 40 in limb motion, interchangeably. After interpretation to data for 3‐month follow‐up, we conclude that good outcomes are more likely to occur in neural anastomosis with nerve conduit than with neurorrhaphy (OR = 5.84, 95% CI = 2.37, 14.42, *p* = .0008). Interestingly, the time course to motor regeneration in neurorrhaphy is often shorter than those in nerve conduit (*p* < .05).

The challenge of restoring limb motor function in subjects with PNI could explain the similar outcomes among groups. The optimal timing for neurotization has been accepted as 3–6 months after loss of limb activities in subjects who have not shown clinical reinnervation, although early reconstitution of neural conduction route in victims has been advocated. A prolonged denervation after limb paralysis can cause irreversible atrophy of target muscle fibers, besides axonal regeneration is known to occur at only approximately 1 mm per day. When considering the difference in timing for the regenerating fibers to reach the end organ, note that rapid neural regeneration is a prerequisite for motor functional recovery. Shortening time period of axon regrowth and improving the percentage of axonal outgrowth seem to be crucial for the flexion and extension. There was no statistical significance in time to axonal regeneration for nerve conduit versus neurorrhaphy (*p* = .53), although our meta‐analysis demonstrated that good outcomes were more common for subjects in neurotube‐dependent anastomosis than in direct suture (OR = 4.8, 95% CI = 1.37, 14.40, *p* = .006 and OR = 2.43, 95% CI = 2.09, 5.94, *p* < .001). There are some reasons why differences in functional recoveries may exist between this two coaptation techniques. Nerve conduit offers a suitable microenvironment that is conducive to axonal regeneration and accurate arrangement. Stump nerves sutured into conduit can be vascularized by infiltration of vessels in surrounding tissues. Furthermore, no barricade from ambient environment is preserved for efficient regeneration with nerve conduit, by which more regenerating axons successfully reach the end organ, and more inputs from motor cortex are delivered to the target muscles to produce the flexion and extension on a paralyzed limb. In comparison, due to lack of autonomous opportunity, either misdirected or mismatched axonal regrowth is observed in end‐to‐end suture, although neurorrhaphy take less time to innervate the axons.

The mechanisms underlying which nerve transfer is used to reanimate paralyzed limbs after PNI are currently explored by researchers. Based on renewability of peripheral nerve roots, paraplegic limbs can be recontrolled by motor cortex ipsilateral to lesion if commands from motor regions are smoothly transported to the end organ by the reconstructed pathway. In attempt to restore continuity of injured nerve in its native status, experienced surgeons often use neurotube to conduct personalized anastomosis depended on size of a nerve gap in repair of PNI. However, further studies are needed to investigate the effect of selected anastomosis on recovering the paralyzed limb after PNI.

The limitations to our study include the neglect of some studies via exclusion criteria that may have provided sufficient subject numbers to demonstrate statistically significant superiority of one technique over another. Notably, the outcome of our analysis and the conclusions of our review account only for subjects who were subjected to neural reconstruction relatively late. The analysis should be interpreted with regard to the interval between paralysis and neurotization (12 ± 1.6 days). The conclusion may be affected by the inconsistency of the articles included in the study. For example, the use of studies recruiting different subjects to support neurotization for motor deficit was rarely reported, and false negatives or positives may have contributed to the surgical decision‐making and thereby the outcomes; another inconsistency was the use of postoperative rehabilitation, which could likewise affect the surgical decision‐making and outcomes. Likewise, the pooling of disparate neural connectivity strategies entailed by the small subject numbers may affect the analysis if any of the individualized anastomosis proves remarkably superior or inferior in the future. The well‐recognized phenomenon that reports focusing on the best outcomes can also affect a systematic review, especially if there is a difference in the number of reports for the various nerve coaptation strategies during a stated time period. However, a randomized, prospective trial will be needed in future analyses when/if systematic reviews are inadequate; significant bias was introduced in this review because it was based on uncontrolled studies. As a conventional therapy, efficacy of allograft in repairing median nerve defect should be evaluated. Finally, the examination of outcomes was performed by different investigators in each study, and the outcomes can be confounded by subjective judgment. Appropriate assessment of subject functional outcomes should be considered to be more than just motor function. Further study into neurotube‐based anastomosis is carried out pathophysiologically and neurobiologically for an overall functional state.

## 5 CONCLUSIONS

Our purpose was to investigate whether a difference in reported outcomes for gap‐specific anastomosis to treat motor deficit existed between neurotube‐dependent coaptation and direct suture. Based on current data, there is an evidence that conduit‐assisted coaptation can dramatically accelerate the motor functional recovery in medium nerve defects (0.5–3.0 cm), while direct suture contributes to motor regeneration in small defects (<0.5 cm). Optimal outcomes will be derived from defect‐guided coaptation. However, large, multicenter RCTs calculating efficacy of individual coaptation in reanimation of the paralyzed limb after PNI are still necessary, especially for a variety of nerve gaps.

## CONFLICT OF INTERESTS

Authors declare no conflict of interests.

## AUTHOR CONTRIBUTIONS

T.Q., C.X., and K.Q. designed the analysis. T.X., J.S., and T.M. collected and extracted the data. T.Q., T.X., and Z.S. carried out the statistical analysis. T.Q. and L.L. drafted the manuscript. All authors reviewed and approved the final report.

## Data Availability

Reasonable requests for data on this study will be reviewed by the corresponding author.
