# Supplementary figures and images for: Efficacy evaluation of personalized coaptation in neurotization for motor deficit after peripheral nerve injury: A systematic review and meta‐analysis
Source: Brain Behav. 2020 Mar 3;10(4):e01582. doi: 10.1002/brb3.1582 (PMC7177589; doi:10.1002/brb3.1582)

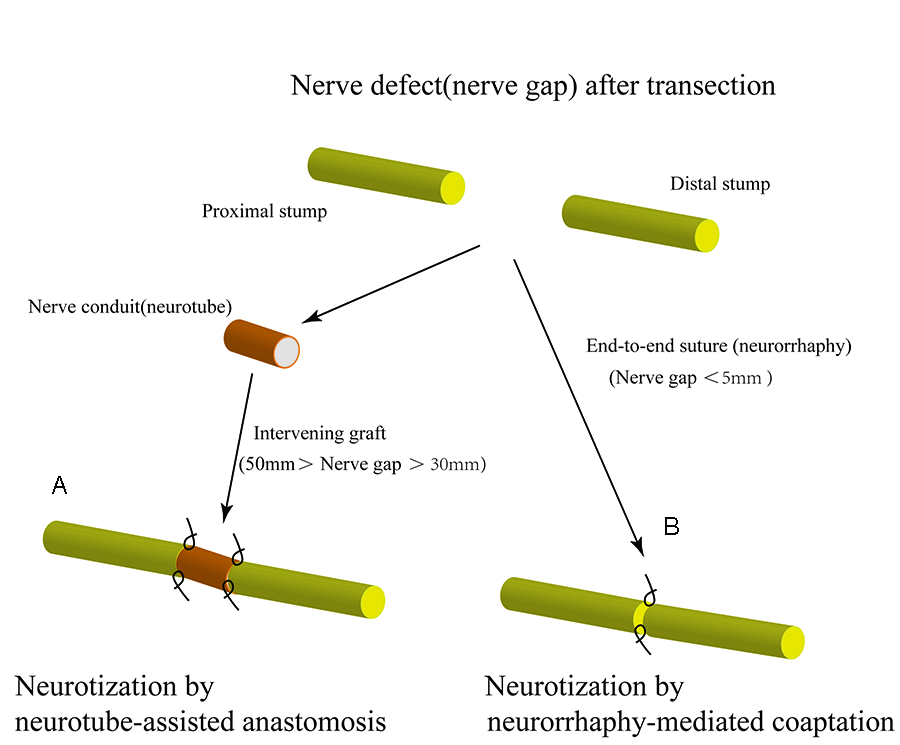

Supplement: Supplementary file 1 [file BRB3-10-e01582-s001.tif]
